# Hepatocellular Carcinoma Bridging and Downstaging: Advances in Locoregional Therapy

**DOI:** 10.3390/biomedicines14040877

**Published:** 2026-04-12

**Authors:** Elliott L. Fite, Nikhil Sekar, Jenish S. Venancius, Mina S. Makary

**Affiliations:** 1 College of Medicine, The Ohio State University, Columbus, OH 43210, USA; elliott.fite@osumc.edu (E.L.F.); nikhil.sekar@osumc.edu (N.S.); jenish.venancius@osumc.edu (J.S.V.); 2 Division of Interventional Radiology, Department of Radiology, The Ohio State University Wexner Medical Center, Columbus, OH 43210, USA

**Keywords:** Hepatocellular carcinoma, locoregional therapies, bridging and downstaging

## Abstract

Hepatocellular carcinoma (HCC) remains a major contributor to global cancer mortality, with many patients presenting beyond the bounds of upfront curative therapy (resection/transplant). Locoregional therapies, particularly transarterial chemoembolization (TACE), transarterial embolization (TAE), and transarterial radioembolization (TARE), therefore play an essential role in bridging and downstaging strategies designed to enable curative intent in otherwise ineligible patients. Bridging therapy aims to maintain transplant eligibility and reduce waitlist dropout, whereas downstaging seeks to reduce tumor burden to meet accepted criteria for resection or transplantation. This review synthesizes current evidence on TACE, TAE, and TARE for bridging to resection and transplantation, as well as for downstaging to surgical eligibility, drawing from systematic reviews and cohort studies in the recent literature. We examine modality-specific outcomes, contextualized by tumor biology, liver function, and treatment selection criteria. Comparative effectiveness and the need for standardized outcome measures will be highlighted, reflecting heterogeneity in study endpoints and patient populations. Finally, future directions in personalized locoregional therapy, integration with systemic therapies, and refined conversion strategies will be discussed, with emphasis on the need for consensus in defining treatment success. By integrating evolving clinical evidence with practical application, this review will help clarify the expanding role of locoregional therapies in enabling curative-intent strategies for HCC.

## 1. Introduction

Hepatocellular Carcinoma (HCC) is the most common type of liver cancer [1,2,3,4] and remains the third leading cause of cancer-related deaths [5,6]. Although life expectancy for patients with HCC has been increasing over the past several years, it still remains low, with a 5-year survival rate of 20% [7]. This low survival rate is likely due to advanced staging at the time of diagnosis. Transplantation and surgery have traditionally been the definitive therapy for HCC, but only select patients are eligible. Less than 20% of patients with HCC meet surgical resection criteria, which is recommended only for patients with adequate liver function, no portal hypertension, and in solitary tumors with no vascular invasion [5,8]. To meet transplant eligibility, patients must satisfy the Milan Criteria (one tumor measuring ≥ 2 cm and ≤5 cm or 2–3 tumors measuring ≥ 1 cm and ≤3 cm and no extrahepatic metastasis or vascular invasion) [9,10,11]. Prolonged transplant waiting times present a significant barrier to treatment for patients with HCC. For these patients, Locoregional Therapies (LRT) represent both bridging strategies to maintain transplant eligibility and downstaging strategies to achieve eligibility in patients initially deemed ineligible [5].

All HCC transplant candidates must wait on the transplant list for 6 months from their initial listing before they can receive their Model for End-Stage Liver Disease (MELD) scoring for transplant [10]. The only exception to this waiting period is for patients who have undergone resection and recurrence that still meets the Milan Criteria [5]. The majority of patients on the waiting list receive some LRT for bridging, but there is not yet an official consensus on which treatment modality is superior [12].

While bridging plays a large role in transplant, it has a slightly different meaning in surgical resection since wait times are often <3 months. Even in instances where surgical wait times are >3 months, oncological outcomes have seemed similar to low wait times in trials [13]. Bridging to resection more often refers to achieving adequate liver volume for a safe resection, termed future liver remnant (FLR) hypertrophy. A higher FLR often indicates a lower chance of post-hepatectomy liver failure and FLR is measured as a percentage of the total liver volume [14].

Downstaging for resection has shown potential benefit, with a meta-analysis showing that a group receiving LRT before resection had increased overall survival (OS) compared to a group receiving LRT/systemic therapy alone: 1 year (RR 1.87, 95% CI 1.48–2.38), 3 years (RR 5.56, 95% CI 2.55–12.10), and 5 years (RR 5.47, 95% CI 2.22–13.49) [15].

As for downstaging to transplant eligibility, the United Network for Organ Sharing Down-Staging (UNOS-DS) has recommended downstaging if there is a single lesion 5.1–8 cm, 2–3 lesions ≤ 5 cm with sum ≤ 8 cm, or 4–5 lesions ≤ 3 cm with sum ≤ 8 cm, without vascular invasion or extrahepatic disease [16]. In addition, patients with initial AFP ≥ 1000 ng/mL must have their AFP decreased to <500 ng/mL to be considered successfully downstaged [5]. Downstaging has shown clear efficacy in clinical trials, similar to those who were originally within Milan Criteria. The XXL trial, which enrolled 74 patients beyond Milan Criteria who were successfully downstaged, showed that those who underwent transplant had an overall survival of 77% versus 31% in the non-transplantation control group (HR 0.32, 95% CI 0.11–0.92, *p* = 0.035) [17].

For patients without curative ability or intent, LRT can also be used for palliative care. For those that have contraindications to LRT or have had progression on LRT, systemic chemotherapy is currently recommended for palliation. Many preliminary trials have looked at a combination approach between systemic therapy and LRT, but this method is not currently recommended as a treatment modality [5].

## 2. Overview of Locoregional Therapies in HCC Management

In patients who are not transplant or resection candidates, or for those where a bridging or downstaging strategy can be applied, LRT is recommended. The broad categorizations of LRT are tumor ablation and transarterial approaches, namely bland TAE, TACE, and TARE [18].

Ablation in HCC utilizes thermal energy to induce tumor necrosis. Radiofrequency ablation (RFA), microwave ablation (MWA), and cryoabalation are widely used. When compared with surgical resection, RFA did not demonstrate any significant difference in overall survival rate or recurrence-free survival for tumors of less than 4 cm [19]. Ablation is actually recommended as a curative therapy in patients with tumors < 3 cm [5,8]. RFA remains well-studied, but MWA has gained increasing use because it can generate higher intratumoral temperatures, create larger ablation zones, and is less susceptible to heat-sink effects [20]. In the pre-transplant setting, retrospective data support MWA as an effective bridging therapy. Som et al. reported histopathologic necrosis in 66% of cases after CT-guided percutaneous MWA, with a median time-to-transplant of 12.6 months [21]. In a separate single-center study, Couillard et al. reported that 80.7% of listed patients ultimately underwent transplant after first-line MWA. HCC-specific dropout was 4.5%, adverse events occurred in 5.1% of patients, and 5-year post-transplant overall survival was 76.7% [22]. Thus, MWA should be considered an emerging pre-transplant ablative option, particularly in patients with small tumors amenable to percutaneous therapy [21,22].

The transarterial therapies are centered around the fact that the liver receives dual blood supply from both the portal vein and hepatic artery. Liver tumors cause angiogenesis and end up being supplied more by the hepatic artery, while the general liver parenchyma can mostly subsist off the portal vein. Bland TAE starves the tumor of its hepatic artery supply, causing ischemia and tumor necrosis [9]. While largely supplanted by TACE and TARE at this point, TAE tends to result in less side-effects from chemotherapy or radiation.

**Figure 1:**
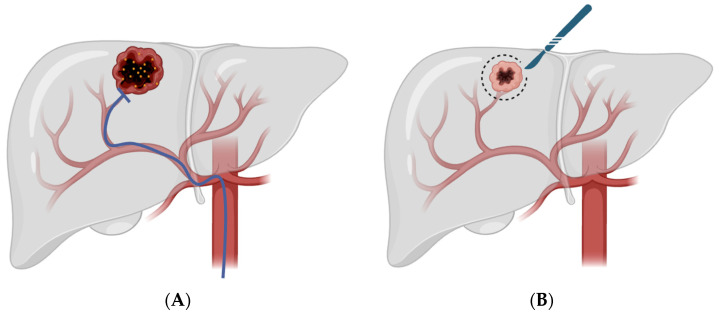
This figure illustrates the use of TACE in downstaging for surgical resection. In panel (**A**), a catheter is inserted through the hepatic artery to both embolize vessels and release cytotoxic agents near the tumor site. Panel (**B**) illustrates surgical resection after the tumor has been successfully downstaged by TACE.

TACE exploits the preferential arterial supply of HCC by delivering embolic material together with chemotherapy through the hepatic artery (Figure 1) [23,24]. A meta-analysis of 14 studies demonstrated an improved 2-year survival compared to control (OR, 0.53, (0.32–0.89)), and with an overall objective response in 35% of patients (16–61%) [25]. Conventional TACE (cTACE) typically uses an emulsion of chemotherapeutic agent and Lipiodol followed by embolication, whereas drug-eluting bead TACE (DEB-TACE) uses calibrated microspheres that allow controlled and sustained intratumoral drug release [25,26,27,28]. In a prospective series of HCC treated with DEB-TACE, Spreafico et al. reported an objective response rate of 77.7%, with a low grade ¾ adverse-event rate of 1 out of 65 procedures [29]. Among patients bridged or downstaged to transplantation or surgery, pathology showed greater than 90% necrosis in 10 out of 28 nodules [29]. Collectively, these data support DEB-TACE as an effective and well-tolerated contemporary embolotherapy platform in HCC.

Comparative evidence suggests cTACE and DEB-TACE have comparable efficacy. A meta-analysis by Facciorusso et al., found that DEB-TACE was not superior to cTACE with respect to tumor response or survival in HCC [30]. A different meta-analysis of randomized trials similarly found no consistent overall survival advantage for DEB-TACE compared to cTACE, although safety and tolerability outcomes differed across studies [31].

TARE utilizes yttrium-90 (Y90) microspheres to deliver high-dose radiation therapy to the tumor vasculature. The 2021 LEGACY trial of 162 patients produced an objective response rate (ORR) of 88.3% (82.4–92.4%) with a three-year overall survival of 86.6% for all patients. This trial resulted in TARE being approved for intermediate-stage HCC as well [5,32,33,34]. TARE has also shown that it can be used safely in cases of portal vein thrombosis since it causes minimal arterial occlusion [35].

## 3. Locoregional Therapies as Bridging Strategies to Resection and Transplantation

LTRs serve a critical role in maintaining candidacy for definitive surgical treatment in HCC. As summarized in Table 1, bridging strategies may be directed toward either hepatic resection or liver transplantation (LT), with distinct therapeutic goals and endpoints. In the resection setting, the objective is conversion to safe hepatectomy through tumor control and augmentation of FLR [9,36,37]. In contrast, transplant bridging focuses on preventing waitlist dropout, maintaining eligibility criteria, and optimizing post-transplant oncologic outcomes [38,39].

### 3.1. Bridging to Transplant

In the transplant setting, the goals of bridging therapy shift from optimizing FLR to waitlist stability, maintenance or downstaging within established Milan criteria, and achieving explant pathologic response, which has emerged as a meaningful surrogate for post-transplant recurrence risk.

Tohme et al. evaluated Y-90 as a sole bridging modality in 20 HCC patients and found the median time from first treatment to transplant was 3.5 months [41]. All patients initially within Milan criteria remained within criteria, and 33% of those initially beyond criteria were successfully downstaged [41]. Complete or partial response determined by mRECIST occurred in 45% of patients, and complete pathologic necrosis was observed in 36% of transplanted tumors that were within Milan criteria at first treatment [41]. Taken together, the data from Tohme et al. support Y-90 as an effective approach for both maintenance of transplant candidacy and selective downstaging.

Radunz et al. extended transplant bridging evidence by correlating explant necrosis with recurrence and survival after Y-90 bridging in 40 transplanted patients [40]. Explant pathology demonstrated complete or partial necrosis in 87.5% of patients overall. Median overall survival was 46 months, and recurrence developed in 9 patients (22.5%) at a median of 15 months after transplant [40]. Recurrence was associated with higher pre- (*p* = 0.0234) and post-treatment (*p* = 0.236) AFP levels and more frequent microvascular invasion (*p* = 0.0163) [40].

In the retrospective study by Hodavance et al., TAE successfully maintained HCC within Milan criteria in 78% of patients at 1 year as a bridging strategy to liver transplantation [45]. The study included 117 patients with HCC meeting Milan criteria at baseline who underwent bland embolization as their initial and sole therapy, with a median time to disease progression beyond Milan criteria of 22.6 months (95% CI, 16.2–29 months) [45]. At 6 months post-embolization, 87% of patients remained within Milan criteria. Ultimately, 29% of patients (*n* = 34) underwent liver transplantation at a median of 3.3 months after embolization, and liver transplantation was a significant independent predictor of longer survival (6.9 years vs. 2.6 years; *p* < 0.001) [45]. The major complication rate within 30 days was 2.6%, including one mortality, demonstrating that bland TAE is a safe and effective bridging modality that compares favorably with other locoregional embolotherapies [45].

A broader modality comparison was provided by Benko et al., who evaluated post-transplant outcomes after TARE versus TACE. Milan criteria were met far less frequently in the TARE group (20.5%) than the TACE group (65.5%, *p* < 0.0001), yet post-transplant recurrence and overall survival were comparable between modalities [42]. On explant, tumor differentiation, microvascular invasion, and necrosis rates were similar; multivariable modeling identified complete tumor necrosis and the absence of microvascular invasion as independently associated with reduced recurrence, while treatment modality itself was not [42]. These findings not only show that TARE and TACE have comparable outcomes when used as bridging therapies, but also that tumor characteristics such as microvascular invasion and tumor differentiation dominate recurrence risk regardless of bridging modality performed.

Prospective evidence for DEB-TACE in transplant was provided by Affonso et al., who compared bridging and downstaging strategies across 200 treated patients [44]. Transplantation occurred more frequently in the bridging cohort (65.9%; *p* = 0.001), while patients in the downstaging group presented with larger, more multifocal tumors and spent significantly longer on the waitlist (median 10.6 months; *p* = 0.028) [44]. Despite these inherent disadvantages, five-year post-transplant overall survival was statistically similar between groups: 73.5% in downstaging versus 72.3% in bridging (*p* = 0.31) [44]. Recurrence-free survival at five years was 62.1% versus 74.8% (*p* = 0.93), and complete radiologic response occurred more frequently in the bridging cohort (*p* = 0.004) [44]. The key takeaway is that successful downstaging within Milan criteria can yield long-term survival outcomes comparable to those of patients who were initially eligible, even when the starting tumor burden is substantially more advanced.

RFA is frequently used as a bridging modality in patients with small, Milan-eligible tumors [43]. DuBay et al. compared RFA to observation and found that RFA achieved a complete radiographic response rate of 83%, supporting its effectiveness as a local tumor control strategy [43]. Patients treated with RFA remained on the waiting list longer than those who received no LRT (median 9.5 months vs. 5 months) [43]. Despite high initial response rates, tumor-specific dropout was not significantly different between groups (21% with RFA vs. 12% without treatment, *p* = 0.11) [43]. Five-year overall survival and tumor-free survival also showed no significant difference between groups [43]. Multivariable analysis showed transplant likelihood and long-term survival were associated with tumor characteristics (AFP, tumor size, and tumor number) rather than RFA treatment of waiting time [43]. These findings suggest RFA functions primarily as a stabilizing intervention that permits safe prolongation of waiting time without adversely affecting post-transplant outcomes.

### 3.2. Bridging to Resection

Vouche et al. first demonstrated that lobar Y-90 radioembolization induces rapid and sustained volumetric changes with significant right lobe atrophy and contralateral hypertrophy evident within 1 month and persisting longitudinally [46]. Median FLR hypertrophy approached 45% at 9 months [46]. Notably, several patients subsequently underwent successful hepatectomy, confirming that radiation-induced hypertrophy can translate into surgical conversion.

Lewandowski et al. showed that in HCC patients undergoing resection after radiation lobectomy with TARE, median FLR increased from 33% pre-treatment to 43% (*p* < 0.01) before surgery [47]. Operative outcomes were favorable, with short hospital stays, transient hepatobiliary toxicity, and substantial tumor response with over 90% of resected tumors demonstrating greater than 50% pathologic necrosis [47]. These findings support the use of LTRs to induce hypertrophy in a clinically meaningful way, translating to safe HCC resections with durable oncologic control.

Gabr et al. expanded evidence in bridging to resection by comparing lobectomy vs. segmentectomy in 31 HCC patients treated with TARE before hepatectomy [48]. Pre-resection FLR hypertrophy was significantly higher after radiation lobectomy (23.3%) compared with segmentectomy (9%, *p* = 0.037) [48]. Pathology demonstrated a strong treatment effect, with 45% of patients demonstrating complete necrosis and an additional 32% of patients demonstrating between 50 and 99% necrosis [48]. Disease control was achieved in all patients prior to surgery and post-resection overall survival was 96% and 86% at 1 and 3 years, respectively [48]. Median recurrence-free survival was 34.2 months [48]. Given the encouraging rates of pathologic necrosis, overall survival, and recurrence-free survival, the results of Gabr et al. support the use of TARE as an effective bridge to hepatectomy in patients with HCC, particularly in the setting of radiation lobectomy.

Bekki et al. directly compared Y-90 lobectomy with portal vein embolization (PVE). Both approaches achieved FLR targets greater than 40%, but hypertrophy was significantly greater with Y-90 (63%) vs. PVE (36%, *p* < 0.01), while tumor response favored Y-90 with 50% complete response [49].

### 3.3. Safety Outcomes for LRTs as Bridging Therapies in HCC

Safety outcomes across bridging studies demonstrate that LRTs are generally well-tolerated, with minimal complications (Table 1). TACE and TAE are associated with low major complication rates, with one study reporting major complication rates as low as 2.6%. TARE similarly demonstrates a favorable safety profile, with predominantly mild adverse events, rare high-grade toxicity, and no evidence of increased perioperative or post-transplant morbidity (Table 1).

### 3.4. Conclusions for LRTs as Bridging Therapies in HCC Treatment

Across both resection and transplant pathways, the studies summarized in Table 1 reveal that LRTs can function as both a holding strategy for transplantation as well as an effective means to optimize patients for definitive surgical care. In resection candidates, LTRs produce measurable FLR hypertrophy and can yield substantial pathologic necrosis at the time of surgery, supporting its role as a bridge to resection in HCC management. In transplant candidates, multiple modalities are capable of maintaining eligibility and achieving meaningful explant necrosis. However, recurrence and survival correlate with markers of tumor biology like AFP, microvascular invasion and tumor differentiation, rather than the specific LRT technique used in bridging therapy. Therefore, LRT should be carefully tailored to individual patients.

## 4. Locoregional Therapies as Downstaging Strategies to Resection and Transplantation

### 4.1. Downstaging to Resection

The systematic review and meta-analysis by Chen et al. evaluated downstaging therapies for converting unresectable HCC to hepatic resection, including TACE as the predominant modality. Across 20 studies, the pooled downstaging rate to resection was 14% (95% CI 0.10–0.17), with significant heterogeneity reflecting variable patient selection and tumor characteristics (Table 2) [15]. Patients who successfully underwent resection after downstaging demonstrated markedly improved survival compared to those receiving locoregional or systemic therapy alone, with pooled overall survival rates of 88% at 1 year, 64% at 3 years, and 42% at 5 years [15]. Disease-free survival rates were 78%, 47%, and 46% at 1, 3, and 5 years, respectively [15]. Predictors of successful downstaging included tumor number, tumor size, absence of portal vein tumor thrombosis, HBsAg status, and AFP level [15]. It was concluded that downstaging may serve as a screening tool to identify patients with favorable tumor biology who might benefit from surgical resection.

Lanza et al. evaluated bland TAE as monotherapy in 230 patients with unresectable HCC, finding that 14% (37 patients) were downstaged during observation and subsequently received percutaneous ablation. The overall cohort demonstrated 1-, 3-, and 5-year survival rates of 84.8%, 38.3%, and 18.7%, respectively, with patients who were downstaged to ablation achieving the best outcomes (Table 2) [50]. This study supports TAE as a viable locoregional option that can convert a subset of initially unresectable patients to candidates for curative-intent ablation [50]. Notably, this aligns with network meta-analysis data suggesting that bland TAE achieves comparable survival to TACE and DEB-TACE for unresectable HCC.

Tzedakis et al. evaluated TARE with yttrium-90 for converting initially unresectable single large HCC (≥5 cm) to surgical resection, finding a 27.7% conversion rate (20 of 72 TARE patients) (Table 2) [51]. Patients successfully converted to resection received significantly higher Y-90 doses compared to those who remained unresectable (211.89 vs. 128.7 Gy, *p* < 0.001) [51]. Postoperative outcomes were similar between TARE-surgery and upfront-surgery patients, and overall survival at 1, 3, and 5 years was comparable (94%, 86%, 55% vs. 83%, 60%, 47%, respectively; *p* = 0.43) [51]. After propensity score matching, TARE-surgery patients demonstrated significantly better overall survival than upfront-surgery patients (*p* = 0.021) [51]. The authors concluded that TARE may serve as a useful downstaging treatment for unresectable localized single large HCC, providing comparable short- and long-term outcomes to readily resectable tumors.

### 4.2. Downstaging to Transplant

The MERITS-LT Consortium study by Mehta et al. represents the first prospective multiregional evaluation of downstaging outcomes for HCC using UNOS downstaging criteria across 209 patients from seven centers [52]. The study demonstrated a successful downstaging rate of 87.7% at 2 years, with TACE (*n* = 132) and Y-90 radioembolization (*n* = 62) showing equivalent efficacy in achieving downstaging, waitlist dropout rates, and probability of transplantation (Table 2) [52]. Post-transplant outcomes were excellent, with 95% survival at 2 years and only 7.9% HCC recurrence, though 42.8% of explants exceeded Milan criteria despite successful radiographic downstaging [52]. The probability of liver transplantation at 3 years was 46.6% after a median of 17.2 months from initial downstaging therapy [52].

Although the study by Lu et al. evaluated percutaneous RFA as a bridge to liver transplantation in 52 consecutive patients with 87 HCC nodules, 10 patients initially exceeded Milan criteria (Table 2) [53]. Complete tumor coagulation was achieved in 85.1% of nodules based on post-ablation imaging [37]. After a mean waiting time of 12.7 months, only 5.8% of patients dropped out due to tumor progression, and 41 patients underwent transplantation with 1- and 3-year post-transplant survival rates of 85% and 76%, respectively, with no HCC recurrence observed [53]. The study demonstrated that percutaneous RFA is an effective bridge to transplantation for patients with compensated liver function and safely accessible tumors, with tumor-related dropout rates and post-transplant outcomes comparing favorably to published controls of patients with early-stage disease.

The study by Berardi et al. evaluated TARE for downstaging intermediate and advanced HCC to transplantation in 214 patients, achieving a 43.9% radiological response rate with 29.9% successfully listed and 22.8% ultimately transplanted (Table 2) [54]. Risk stratification using tumor burden beyond up-to-seven criteria, AFP > 400 ng/mL, and albumin-bilirubin ≥ 2 identified three prognostic groups with median survivals of 3 years, 1.9 years, and 9 months respectively [54]. Among transplanted patients, 5-year overall survival was 61%, though only 11.5% remained alive without transplant at 5 years, with 73% dying without transplant [54]. The study demonstrates that while TARE enables downstaging in select patients, tumor and patient characteristics are critical for predicting successful outcomes.

The Xie et al. meta-analysis compared TARE and TACE as downstaging or bridging strategies for HCC before liver transplantation, including 12 studies with 10,661 patients. The analysis demonstrated no significant differences between TARE and TACE in downstaging rate (OR 0.96, *p* = 0.88), transplantation rate (OR 0.89, *p* = 0.47), or recurrence rate (OR 1.26, *p* = 0.45) (Table 2) [55]. However, TARE was associated with fewer treatment sessions (*p* = 0.0002), lower grade 3/4 bilirubin toxicities (*p* = 0.03), higher complete tumor necrosis rates (OR 2.16, *p* = 0.02), and significantly better recurrence-free survival (OR 2.39, *p* = 0.03) [55]. The authors concluded that while both modalities demonstrate comparable efficacy in downstaging patients to transplant, TARE may offer practical clinical advantages due to enhanced oncologic efficacy, fewer treatment sessions, and a more favorable safety profile.

Table 3 summarizes comparative studies evaluating TACE and TARE specifically in the transplant setting. Across these analyses, both modalities demonstrate comparable post-transplant OS and recurrence outcomes, supporting their roles as effective bridging therapies (Table 3). However, differences emerge in pre-transplant endpoints. TARE has been associated with lower waitlist dropout and improved tumor response prior to transplantation, suggesting a potential advantage in maintaining transplant eligibility [55,56,57,58]. Despite these findings, the available evidence is largely derived from retrospective and non-randomized studies, limiting any definitive conclusions regarding superiority or either TACE or TARE. Overall, these data support that both TACE and TARE are viable bridging strategies, with modality selection best guided by tumor burden, liver function, and patient specific factors.

### 4.3. Safety Outcomes for LRTs as Downstaging Therapies in HCC Treatment

Safety reporting in downstaging studies remains inconsistent, with several large cohorts lacking standardized documentation of treatment-related toxicity. Among studies reporting outcomes, TARE demonstrates a favorable safety profile, with low complication rates and predominantly mild adverse events, including a 6.5% complication rate and 0.5% treatment-related mortality in a large cohort (Table 2). Meta-analytic data further support reduced hepatic toxicity with TARE compared to TACE, with significantly lower rates of grade 3/4 bilirubin elevation (Table 2) [55]. Additionally, comparative surgical series demonstrate no increase in perioperative morbidity following TARE-based downstaging (Table 2) [51]. Despite these encouraging findings, the limited reporting of safety outcomes represent a significant gap in the current literature.

### 4.4. Conclusions for LRTs as Downstaging Therapies in HCC Treatment

LRTs including TACE, TAE, and TARE demonstrate meaningful efficacy in downstaging initially unresectable HCC to both surgical resection and liver transplantation, with up to 88% for transplant eligibility depending on patient selection [15]. Patients successfully downstaged to curative-intent therapies achieve substantially improved long-term outcomes, with 5-year overall survival rates of 42–55% after resection [52] and 61% after transplantation [54], compared to significantly worse outcomes with locoregional or systemic therapy alone. Meta-analytic data comparing TARE and TACE for downstaging to transplant demonstrate equivalent efficacy in downstaging rates, transplantation rates, and recurrence, though TARE offers practical advantages including fewer treatment sessions, lower hepatotoxicity, and improved recurrence-free survival. Predictors of successful downstaging consistently include tumor burden, AFP levels, and underlying liver function, emphasizing that downstaging serves not only as a therapeutic strategy but also as a selection tool to identify patients with favorable tumor biology. Collectively, these findings support the integration of locoregional therapies as a cornerstone of multidisciplinary HCC management, enabling curative-intent treatment in a substantial proportion of patients who would otherwise be limited to palliative options.

## 5. Future Directions

Several angles can dictate future directions for the utility of locoregional therapies in downstaging and bridging. First, a more concrete definition of “successful downstaging” must be agreed upon since the criteria seems to be heterogenous among studies. In the MERITS-LT study, for example, 42.8% of explants exceeded Milan criteria despite radiographic downstaging success [52]. This paradigm illustrates there is a flawed definition of downstaging success that makes it difficult to separate treatment efficacy from the variable criteria in patient selection. A lack of standardization needs to be agreed upon before conducting reliable comparative studies.

Most of the studies reviewed were single-arm studies. None of the studies directly comparing LRT modalities were randomized control trials (RCT), and there have not been studies comparing every combination of LRT that was discussed. More comprehensive and advanced research comparing TACE, TARE, and ablation should be completed to reliably create recommendations. When comparing modalities, cost-effectiveness and patient satisfaction should also be considered in future studies in addition to survival outcomes.

Biomarkers should also be incorporated into studies to predict downstaging success or post-transplant outcomes. An AFP decrease from >1000 to <500 is a current requirement of a successful downstage, and many of the above studies have listed AFP or albumin [5,52,53] as prognostic indicators for transplant success. These could be studied in relation to various LRT methods to inform guidelines for bridging and downstaging.

Future analyses should also target combination approaches between LRTs and Immune Checkpoint Inhibitor (ICI) treatment. Trials remain promising for this integrative approach; the VITALITY Study, which included 117 HCC patients undergoing liver transplantation assessment, demonstrated 75.6% of patients beyond Milan criteria were successfully downstaged within a median of 5.6 months after pre-treatment with ICI and LRT [59]. A single-center retrospective study of 104 patients that compared an LRT group to a combination group, however, showed a similar time to post-transplant tumor recurrence between both groups (496.8 days vs. 546 days; *p* = 0.41) [60,61,62,63]. With further research, this combination approach has the potential to expand the pool of candidates amenable to transplant or resection [60,64,65,66,67].

## 6. Conclusions

In this review, studies have demonstrated the utility and efficacy of LRT as bridging and downstaging strategies before resection and transplantation for HCC. As bridging strategies, these modalities seem to effectively maintain tumor control in patients initially eligible for curative therapy. The efficacy of LRT in bridging to transplantation is supported by the high rates of radiographic and pathologic necrosis, maintenance of Milan criteria, and favorable post–liver transplant survival rates. Similarly, significant FLR and high rates of tumor necrosis facilitate safe and timely bridging to resection in selected patients.

The evidence base also suggests that downstaging also has similar transplant and post-transplant survival rates compared to patients who were initially within criteria. Likewise, downstaging to resection showed similar outcomes to readily resectable tumors.

Importantly, comparative data thus far have not demonstrated a significant difference between the different LRT modalities in rates of tumor recurrence, downstaging rate, and transplant rate. It seems that monitoring pre-transplant or bridging tumor burden with tumor markers like AFP, for example, play a larger role in determining outcomes. However, more higher-level studies that compare different LRT modalities such as randomized control trials need to be completed before concluding they are equally efficacious. In addition, definitions of downstaging must be standardized in order to properly study comparative modalities.

## Figures and Tables

**Table 1:** Outcomes for LTRs used as bridging therapies for HCC. RFS: recurrence-free survival.

	Study	LRT	Key Outcomes	Safety
Bridge to Transplant	Radunz et al. [40]	TARE	87.5% of patients demonstrated at least partial tumor necrosis; 33% of patients downstaged; 36% complete pathologic necrosis	No waitlist dropout or deterioration in liver function; MELD scores remained stable following treatment
	Tohme et al. [41]	TARE (Y-90)	Maintained Milan eligibility in all patients initially within criteria; 45% had complete/partial response by mRECIST; 36% had complete pathologic tumor necrosis	3 hospitalizations within 90 days after treatment; overall low morbidity
	Benko et al. [42]	TARE vs. TACE	Median OS 35.8 months. Time from LT to tumor recurrence was 14 months. No significant difference in rates of tumor recurrence between TACE and TARE.	No significant difference in post-transplant outcomes
	DuBay et al. [43]	RFA	83% of patients demonstrated complete radiographic response. No difference in 5-year post-LT survival vs. no therapy.	Two minor complications; no major complications or treatment-related deaths reported
	Affonso et al. [44]	TACE	5 year post LT OS: 72.3%; RFS 74.8%	Not reported
	Hodavance et al. [45]	TAE	87% of patients at 6 months, and 78% at 12 months maintained Milan criteria; 29% were able to undergo LT, with a low major complication rate of 2.6%.	Major complication rate 2.6% (including one death); overall complications included 11 minor and 3 major events
Bridge to Resection	Vouche et al. [46].	TARE (radiation lobectomy)	Early and sustained FLR hypertrophy; median 45% FLR at 9 months; enabled hepatectomy and transplantation in selected patients	Only minor adverse events reported (fatigue, abdominal pain, nausea); no major complications observed
	Lewandowski et al. [47].	TARE (radiation lobectomy)	Median %FLR hypertrophy of 30%; 92% of resected tumors had >50% pathologic necrosis	No major complications or new grade 3–4 toxicities; predominantly mild adverse events
	Gabr et al. [48].	TARE	Median 2.9 months to resection; 23% FLR hypertrophy; 45% complete necrosis; 1-yr survival 96% and 3-yr survival 86%.	One case (3%) of grade 3 bilirubin toxicity; no other grade 3/4 toxicities or radiation-induced complications reported
	Bekki et al. [49].	TARE vs. PVE	Greater hypertrophy with Y-90 (63% vs. 36%, *p* < 0.1)	Post-therapy complication rates 9% (TARE) vs. 14% (PVE), most commonly abdominal pain; rare serious complications reported

**Table 2:** Outcomes for LTRs used as downstaging therapies for HCC.

	Study	LRT	Key Outcomes	Safety
Downstage to Resection	Chen et al. [15]	TACE	Across 20 studies, the downstaging rate to resection was 14% (95% CI 0.10–0.17) using TACE.	Safety outcomes not specifically reported
	Lanza et al. [50]	TAE	14% of patients were downstaged and received percutaneous ablation with 1, 3, and 5-year survival of 84.8%, 38.3%, and 18.7%, respectively. Patients receiving combined TAE with RFA had best outcomes.	Safety outcomes not specifically reported
	Tzedakis et al. [51]	TARE (Y-90)	Converted initially unresectable single large HCC (≥5 cm) to surgical resection, finding a 27.7% conversion rate (20 of 72 TARE patients).	Postoperative complication rates were comparable between TARE-to-resection and upfront surgery groups; TARE-specific toxicity not systematically reported
Downstage to Transplant	Mehta et al. [52]	TACE	87.7% of patients at 2 years showed successful downstaging to the Milan Criteria, with patients having a LT probability of 46.6% at 3 years.	Safety outcomes not specifically reported
	Lu et al. [53]	RFA	41 of 52 patients underwent LT with a post-LT 1-year survival of 85%, and a 3-year survival of 76%.	Safety outcomes not specifically reported
	Berardi et al. [54]	TARE	43.9% of patients in this retrospective multicenter study showed radiological response.	Low complication rate: 6.5% overall; 0.5% mortality (liver failure); predominantly mild adverse events (abdominal pain 2.3%, duodenal ulcer 0.9%)
	Xie et al. [55]	TARE vs. TACE	Demonstrated no difference in downstaging rate (OR 0.96, *p* = 0.88), transplant rate (OR 0.89, *p* = 0.47), or recurrence (OR 1.26, *p* = 0.45).	Lower grade 3/4 bilirubin toxicity vs. TACE (OR 0.32); overall favorable safety profile

**Table 3:** Comparative outcomes of TACE and TARE before transplant.

Study	Study Type	Population	Key Outcomes
Xie et al. [55].	Systematic review and meta-analysis	Bridging/downstaging to LT	No difference in downstaging rate, transplant rate, or recurrence; TARE associated with fewer sessions, lower bilirubin toxicity, and improved recurrence-free survival
Zori et al. [56].	Comparative cohort study	HCC patients undergoing bridging to transplantation	No significant difference in overall survival or recurrence between TACE and TARE; treatment modality did not predict outcomes
Lopez-Lopez et al. [57].	Systematic review	Unresectable and intermediate-stage HCC	TARE associated with improved time-to-progression and tumor control; no significant difference in overall survival
Kim et al. [58].	Retrospective comparative study	HCC patients awaiting liver transplant	TARE associated with lower waitlist dropout and improved tumor response compared to TACE; post-transplant outcomes comparable between modalities

## Data Availability

No new data were created.

## Abbreviations

The following abbreviations are used in this manuscript:

TACE	Transarterial Chemoembolization
TARE	Transarterial Radioembolization
HCC	Hepatocellular Carcinoma
RFA	Radiofrequency Ablation
TAE	Transarterial Embolization
LRT	Locoregional Therapy
DEB-TACE	Drug-eluting Bead TACE
PVE	Portal Vein Embolization
